# Low Carb Program Health App Within a Hospital-Based Obesity Setting: Observational Service Evaluation

**DOI:** 10.2196/29110

**Published:** 2021-09-23

**Authors:** Petra Hanson, Charlotte Summers, Arjun Panesar, Dominic Oduro-Donkor, Maria Lange, Vinod Menon, Thomas M Barber

**Affiliations:** 1 Warwick Medical School University of Warwick Coventry United Kingdom; 2 Warwickshire Institute for the Study of Diabetes, Endocrinology and Metabolism University Hospitals Coventry and Warwickshire Coventry United Kingdom; 3 DDM Health Coventry United Kingdom

**Keywords:** obesity, low carb program, eHealth, mobile app, digital health, health intervention, mobile health, COVID-19

## Abstract

**Background:**

Obesity underlies much chronic disease. Digitalization of obesity management provides an opportunity to innovate our traditional model of health care delivery within this setting, and to transform its scalability potentially to the population level.

**Objective:**

The objective was to assess the feasibility and effectiveness of the Low Carb Program app for weight loss, applied within our hospital-based (tier 3) obesity service. Due to the disrupting effects of the COVID-19 pandemic on our obesity service, we compared the clinical outcomes from the Low Carb Program app applied in the context of remote patient appointments over the telephone with the prepandemic traditional standard of care.

**Methods:**

We invited patients who attended our hospital-based obesity service to engage with the Low Carb Program smartphone app. We combined this approach with remote delivery (over the telephone) of obesity management from medical and psychology members of our obesity team during the COVID-19 pandemic. Outcome variables included changes in body weight and changes in HbA_1c_ as a marker of glycemic control. We compared data from the Low Carb Program group with a retrospective control group (n=126) that had received traditional face-to-face obesity management from our team without concomitant use of the Low Carb Program app in the pre–COVID-19 era. *T* test comparisons were employed, with *P*<.05 considered significant.

**Results:**

The mean weight of participants (n=105) was 130.2 kg, with 59% (n=62) females and a mean age of 48.8 years. Most participants (90/105, 86%) completed the Low Carb Program app registration process and engaged with the Low Carb Program app program; at follow-up, most participants (88/105, 84%) had actively engaged with the Low Carb Program app within the prior 30 days. The majority of participants (58/105, 55%) self-reported outcomes within the app. Mean duration of clinical follow-up for recruited participants who received the app was 7.4 months. Paired data were available for 48 participants for body weight and 41 participants for HbA_1c_. Paired sample *t* test analysis revealed a statistically significant mean loss of body weight of 2.7 kg (*P*=.001) and improvement in HbA_1c_ of 3.3 mmol/mol (*P*=.01). The mean weight of control group patients (n=126) was 137.1 kg, with 74% (93/126) females and a mean age of 44.4 years. The mean follow-up for this group was 6 months. Data comparisons between the app user group and the pre–COVID-19 retrospective control group revealed equivalence for loss of body weight and change in HbA_1c_ between the two groups.

**Conclusions:**

We provide evidence to support the feasibility of implementing the Low Carb Program app combined with remote management; this is the first proof of concept for digitalized management within a hospital-based (tier 3) obesity service. We demonstrate the potential clinical efficacy of the approach in terms of improvements in body weight and glycemic control.

## Introduction

In the United Kingdom, 27% of men and 30% of women live with obesity [1] and have an increased risk of chronic diseases such as type 2 diabetes (T2D), mental health problems, and certain malignancies, and a reduced life expectancy [2]. Moreover, people living with obesity have a greater risk of hospitalization and adverse outcomes (including mortality) from COVID-19 [3]. Within the United Kingdom alone, the estimated health care cost of obesity management was £6.1 billion (US $8.5 billion) in 2014—projected to reach £9.7 billion (US $13.5 billion) by 2050—with the wider costs to society estimated at £49.9 billion (US $69.2 billion) per annum [4].

Within the UK-based National Health Service (NHS), a tiered framework underlies the pyramidal structure of obesity management, in which progressively restricted resources and numbers of patients typifies upward tier progression. Obesity management within a primary care setting (tier 1) consists of promoting healthy lifestyles, education, and preventive strategies. Community-based services (tier 2) usually comprise dietary advice and optimized physical activity. For people with more severe obesity (BMI>40 kg/m^2^ or BMI>35 kg/m^2^ with obesity-related comorbidities), there is hospital-based obesity management (tier 3), which usually consists of a multidisciplinary team, with specialist dieticians, medical staff, and psychological support. Bariatric surgery (tier 4) sits at the top of the structure, and as such represents a restricted resource (despite representing an excellent treatment choice for obesity) available to a very small proportion of the obese population who are potentially eligible for this procedure [5].

Hospital-based management of obesity (within tiers 3 and 4) is restricted, and as such does not represent a scalable model applicable to a population level [6]. Furthermore, the demand for obesity services including referrals has augmented in recent years. We illustrate this with the example of our own hospital-based tier 3 and 4 obesity service at University Hospitals Coventry and Warwickshire (UHCW), United Kingdom, with a >6-fold increase in the number of referrals over a 5-year period between 2014 (207 new referrals) and 2019 (1319 new referrals), and >1800 patients currently accessing our obesity service. Despite this surge in new referrals, this number is likely an order of magnitude lower than that of local adults who are eligible for referral to our service, with an estimated 2% of men and 4% of women currently living in the Coventry and Warwickshire region who have a BMI>40 kg/m^2^ [7-9]. Thus, there is a large unmet need for the vast majority of people living with severe obesity in the United Kingdom, for whom the current hospital-based tier 3 and 4 services within the NHS obesity management framework simply fails to deliver. Furthermore, there is often a disjointed patient pathway between the various obesity management tiers within primary, community-based, and secondary care settings. Unfortunately, the COVID-19 pandemic has stymied the effective implementation of obesity management across all tiers, with multiple factors implicated including staff redeployments, repurposing of clinical areas, restrictions of elective procedures, lockdown measures, remote appointments, and patient-based fears that include attendance at health care settings.

The NHS Long Term Plan has set a goal of developing digital interventions to improve patient outcomes and effectiveness of care [10]. However, despite the potential utility of various apps for digital and mobile devices within hospital-based tier 3 and 4 obesity management settings, to date such an innovation has not occurred. There is emerging evidence for the effectiveness of digital tools in the management of obesity. In China, a digital weight loss program was tested in >8000 people with obesity (BMI≥35 kg/m^2^) in a retrospective observational analysis [11]. The program consisted of the implementation of an app for a mobile digital device, daily self-weighing, and meal replacements. Users who engaged with the digital intervention lost 8.1 kg of body weight over 42 days [11]. Unfortunately, the lack of a control group limited interpretations of the efficacy of the mobile app per se versus confounders such as meal replacements. A systematic review of 17 randomized controlled trials demonstrated the effectiveness of apps for digital mobile devices for effecting weight loss (greater effect on weight loss than nondigital weight loss interventions) [12]. Apps for digital mobile devices may improve the behavioral determinants of obesity (such as dietary intake), and enable the continuous targeting of healthy behaviors related to weight management [13] and the maintenance of healthy behaviors with minimal professional input [14]. Edson and colleagues [15] evaluated the efficacy of a “digital lifestyle change” program for the improvement of body weight in people with obesity, and demonstrated that engagement with a 6-week program was associated with a mean weight loss of 8.2% at 6 months. However, the data were obtained from only 15 participants, including those who self-funded the intervention, with a lack of feasibility data. Finally, a recent meta-analysis on 6 systematic reviews demonstrated the association of apps for digital mobile devices with a weight loss of 1-2.4 kg and improved glycemic control (HbA_1c_ reduction of 0.3%-0.5%) [16].

The Low Carb Program is a multiplatform, NHS-approved digital health intervention that supports weight loss through the provision of therapeutic carbohydrate restriction, intended for people living with obesity, prediabetes, or T2D [17]. The app is available on iOS, Android, web, smartwatch, smart speaker, and virtual reality platforms. An evaluation of the Low Carb Program app among 1000 people with T2D who self-reported their outcome measures (online recruitment and use of the app for 1 year) showed that engagement with the app resulted in a mean weight loss of 7.4 kg (7% reduction in body weight from baseline). There was also improved glycemic control (HbA_1c_ reduction of 13 mmol/mol) and remission of T2D in 26% of the participants, sustained at 1 year [17]. However, despite such promising early data, no assessment of the Low Carb Program app has yet occurred within a hospital-based tier 3 obesity service to date.

The aim of our study was to assess the feasibility and effectiveness of the Low Carb Program app for weight loss, applied within our hospital-based (tier 3) obesity service. Due to the disrupting effects of the COVID-19 pandemic on our obesity service, we also compared the clinical outcomes from the Low Carb Program app applied in the context of remote patient appointments over the telephone with the prepandemic traditional standard of care.

## Methods

### Participants

**Figure 1:**
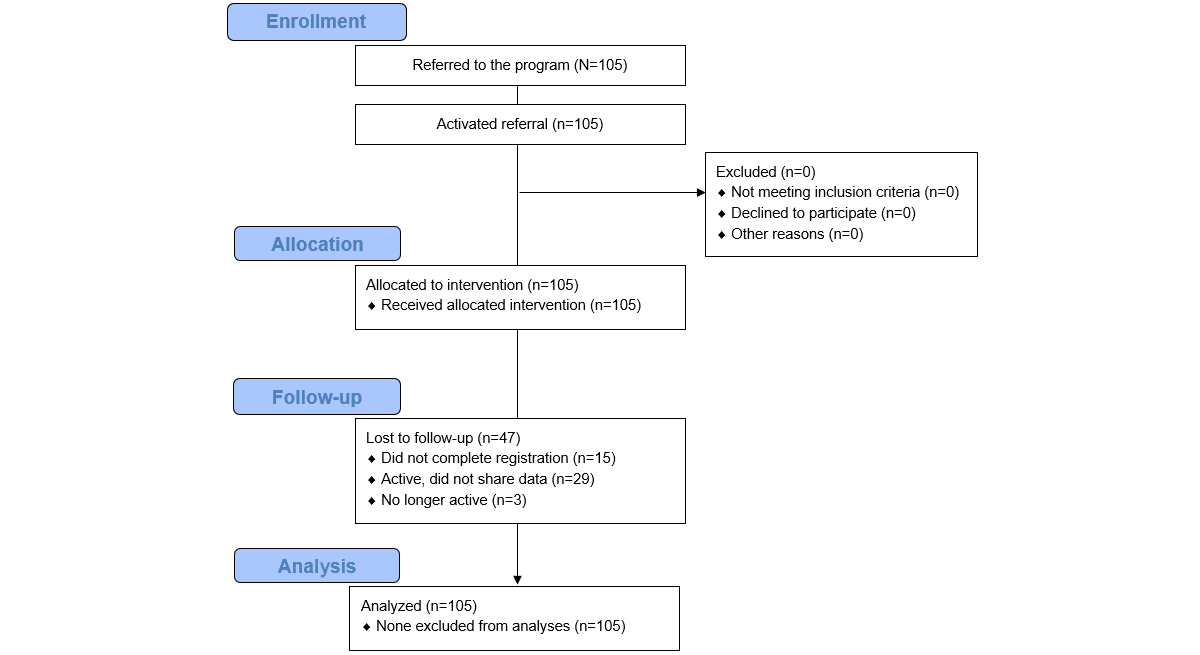
CONSORT diagram.

Access to the Low Carb Program app was offered to all new patients at their initial medical consultation after being referred to our hospital-based (tier 3) obesity service at UHCW, United Kingdom, over a 9-month period between January 2020 and September 2020. The initial contact was with a medical doctor. Patients were informed about the app and those who wanted to use the app were given instructions on how to download it. We recruited those patients who were interested in using the app (n=105), and provided each of them with a unique code that enabled activation of the app free of charge when downloaded from the NHS App Library. The only exclusion criterion included inability to understand English. Figure 1 summarizes the journey for study participants. The control group data were collected for a previous study assessing group educational sessions, with the participants attending the service between 2016 and 2019. The duration of follow-up for the control group was 6 months.

### Research Design

Due to the observational nature of our study, and its innovation as a strategy to improve clinical care, no formal approvals from research ethics or institutional review board committees were required. We registered our study as a service evaluation with the Research and Development team at UHCW. We integrated provision of the Low Carb Program app to patients as part of their obesity management. As such, it was not possible to randomize this intervention. Aligned with their clinical care, participants did not receive any payment for their participation in our study. Participants provided their consent for use of their anonymized self-reported data from the Low Carb Program app for research purposes. Inclusion in the study and overall engagement with the Low Carb Program app did not affect patients’ clinical management.

### Low Carb Program App

The Low Carb Program app is an NHS-approved medical device (“Software as a Medical Device”) that provides a 12-week digital health intervention with personalized and structured content for adults diagnosed with T2D, prediabetes, and obesity [18]. The Low Carb Program app provides the user with access to weekly nutrition-focused educational modules, supported by action points and suggested stepwise changes for the user to execute over the subsequent week. The Low Carb Program app module design enables participants to gradually reduce their total daily carbohydrate dietary intake to <130 grams, with a focus on increased consumption of unprocessed foods (including home cooking and food preparation from raw ingredients) and reduced consumption of processed and ultraprocessed foods, to address self-selected health goals. The Low Carb Program app also provides the user with downloadable and printable resources that include information sheets, recipes, meal plans, and suggested food substitution ideas. The Low Carb Program app also includes digital tools for the user to submit their self-monitoring data (via connection of their wearables to the Low Carb Program online platform) on diverse variables that include blood glucose levels, blood pressure, mood, sleep, diet, and body weight. There is provision of individualized weekly feedback to participant users based on their use of the Low Carb Program app, delivered via email and in-app push notifications. Lessons are adapted to different learning styles, delivered through videos, written content, and podcasts of varying lengths (ranging from 3 to 12 minutes in duration). The app is currently used within NHS primary care and provides native language support for “hard-to-reach” communities of Punjabi, Hindi, Gujarati, Urdu, and Bengali speakers.

For the purposes of our study, we used a streamlined version of the Low Carb Program app, specifically tailored to people living with obesity. In the first 2 weeks of the Low Carb Program app program, participant users receive education on the physiology of obesity and the role of dietary modification in its management. This includes a description of how a low-carbohydrate diet can improve the management of postprandial blood glucose levels (in T2D) and body weight. The subsequent Low Carb Program app modules explore strategies to reduce dietary sources of sugar, including foods with a high starch content, such as bread, pasta, potatoes, and rice, and foods that contain refined sugar. Participants are encouraged to make portion control and carbohydrate restriction decisions based on visual plate representations. In place of carbohydrate-rich foods, the Low Carb Program app modules advocate an increased intake of green vegetables, low glycemic index fruits (blueberries, strawberries, and raspberries) and healthy fats (including olive oil, butter, eggs, nuts, and dairy products). Participants could access the tools supplied within the Low Carb Program app, including educational modules, and were encouraged to track their body weight and other measures of overall health, and access support from the community discussion board. Participants also had access to a searchable library of recipes tailored to dietary preferences and allergies. Digitally excluded patients are supported with a physical “starter pack” containing a welcome booklet, educational modules and resources, and cookbook composed of recipes from the program. Digitally excluded patients are encouraged to join weekly coaching and meetup sessions that can be attended by telephone. We provide a list of the weekly educational topics for the Low Carb Program app in Table 1.

**Table 1:** Core syllabus of the Low Carb Program.

Lesson	Topic	Objective
1	Welcome to the Type 2 Diabetes Program	Safety notes and alerts to medications that require health care professional team’s assistance Benefits of a reduced carbohydrate diet for people with type 2 diabetes, prediabetes, and obesity
2	Blood glucose levels and diet	Factors that affect blood glucose levels Encouragement to engage with their health care providers
3	Controlling portion sizes	Introducing visual methods for interpreting portion size
4	Real vs processed foods	Identifying and eliminating refined and processed food
5	Healthy and unhealthy fats	Discussion of fat types and making appropriate choices depending on goals
6	Vegetables	Demonstrating the carbohydrate content of vegetables and cooking methods
7	Fruit	Reviewing the amount of sugar and starch in fruit and vegetables
8	Snacks and desserts	Examining low-carbohydrate snack, dessert, and drink options
9	Drinks	Tips on alcohol and eating out options
10	Eating out and takeaways	Managing eating on the go and when travelling Making healthier takeaway and food choices
11	Practical ways to eat fewer carbohydrates	Practical tips for reducing carbohydrate intake further Safety information, highlighting medications that require health care provider assistance
12	Intermittent fasting	Introducing the principles of reducing the eating window using the 16:8 model

### Follow-up

Each recruited participant had ongoing clinical input and follow-up with members of our hospital-based (tier 3) Obesity management team as part of usual care throughout the study period. Although conceived and commenced within the pre–COVID-19 era (January 2020), there was substantial overlap of much of our study with the COVID-19 pandemic (for 5 of the 7 months of the study period, between March and September 2020). As a result, no patient in the tier 3 weight management service received specialist dietary input from March 2020 onward. The clinical follow-up varied between patients but most received telephone review by a doctor 6 months after the previous appointment. However, the Low Carb Program app supported each participant with invited virtual meetups every Monday to provide an opportunity for social connection with other users during the “lockdown” period in the United Kingdom. We conducted the virtual meetups through coach-led video conferencing sessions that provided an informal space for the sharing of personal experiences and establishment of peer support networks.

### Statistical Analyses

The total sample size required to detect an expected standardized difference Δ at two-sided significance level *α* and power 1–*β* is given by the following expression [19]:




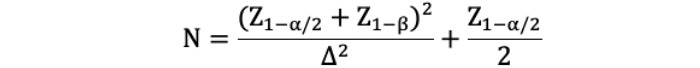




Where Z_1–*α*/2_ and Z_1–*β*_ are percentage points of the normal distribution, which for 5% significance and 80% power are given by Z_1–*α*/2_=1.96 and Z_1–*β*_=0.84 with standardized difference Δ=(d_t_–d_0_)/sd, whereby sd is the standard deviation of the difference, and d_t_ and d_0_ are a group mean at time *t* and baseline, respectively. An approximate expression is given by N ≈ 2+8/Δ^2^ for 80% power at the 5% level.

The study was planned based on a clinically significant expected change in mean weight (weight loss) of 1 kg at time point *t*. A large cohort study on outcomes of a specialist weight management program in the United Kingdom found that the mean weight loss of 1838 patients was just under 1 kg at 3 months [20]. The standard deviation of the participant weight in this population is unknown, but we expected a large to moderate sized standardized mean difference, which suggests values in the range of 0.5 to 1. A sample size of 10 participants will provide 80% power to detect a change in weight of 1 kg. Therefore, a sample size of 48 patients with matched weight was adequate to detect a clinical effect if it existed. Recruiting 10 individuals into the study provided reasonable power to detect a clinically significant effect in weight loss. A power calculation for changes in HbA_1c_ was not done.

Statistical analyses were performed using SPSS (version 27.0; IBM Corp). The primary outcome variables were change in body weight and HbA_1c_ (measured as part of routine clinical care) and user engagement with the Low Carb Program app. Quantile-quantile (Q-Q) plots were used to test the assumption that data on body weight were normally distributed. Data on HbA_1c_ and age were assumed to be normally distributed. We compared baseline and follow-up data from the Low Carb Program app users through the paired Student *t* test, with a *P* value of <.05 considered significant. For comparison of the efficacy of the Low Carb Program app to the pre–COVID-19 pandemic standard of care, we obtained retrospective control data collected between 2016 and 2019 from a random selection of patients (n=126) attending our UHCW-based obesity service during that time. Comparison of changes in body weight and HbA_1c_ between the retrospective control group and the Low Carb Program app user group were compared using the independent Student *t* test, with a *P* value of <.05 considered significant. We obtained data regarding user engagement with the Low Carb Program app (including Low Carb Program app activation and duration of use) from Diabetes Digital Media. Chi-square test was used to compare baseline data (gender and percentage of patients with diabetes mellitus) from the intervention group to the retrospective control group.

## Results

### Baseline Participant Characteristics

We provide data on baseline characteristics of the Low Carb Program app users (n=105) in Table 1. These include a mean baseline body weight of 130.2 (SD 29.2) kg, a mean age of 48.8 (SD 12.7) years, and a mean HbA_1c_ of 48.0 (SD 15.5) mmol/mol. The majority of participants (n=62, 59%) were female. Overall, a minority of participants (n=38, 36.9%) had diabetes mellitus (type 1 diabetes: n=5; T2D: n=33). Baseline weight and HbA_1c_ data were missing from 2 Low Carb Program app users. The baseline phenotype of Low Carb Program app users was broadly similar to the retrospective control group in terms of baseline weight, proportion of patients with diabetes, and baseline HbA_1c_, but was significantly different in terms of age and gender (Table 2).

**Table 2:** Baseline characteristics of patients included in the Low Carb Program app group (combined with remotely delivered obesity management) versus retrospective control participants who received a traditional standard of care delivered before the COVID-19 pandemic.

Characteristic		Low Carb Program participants (N=105)	Control group (N=126)	*P* value
Age (years), mean (SD)		48.8 (12.7)	44.4 (13.3)	.01
HbA_1c_ (mmol/mol), mean (SD)		48.0 (15.5)	45.3 (14.3)	.13
Weight (kg), mean (SD)		130.2 (29.2)	137.1 (27.0)	.07
**Gender, n (%)**				
	Male	46 (43.8)	52 (41.3)	0.02
	Female	59 (56.2)	74 (58.7)	0.02
**Health conditions, n (%)**				
	Diabetes mellitus	38 (36.2)	41 (25)	0.06

### Low Carb Program Engagement

**Figure 2:**
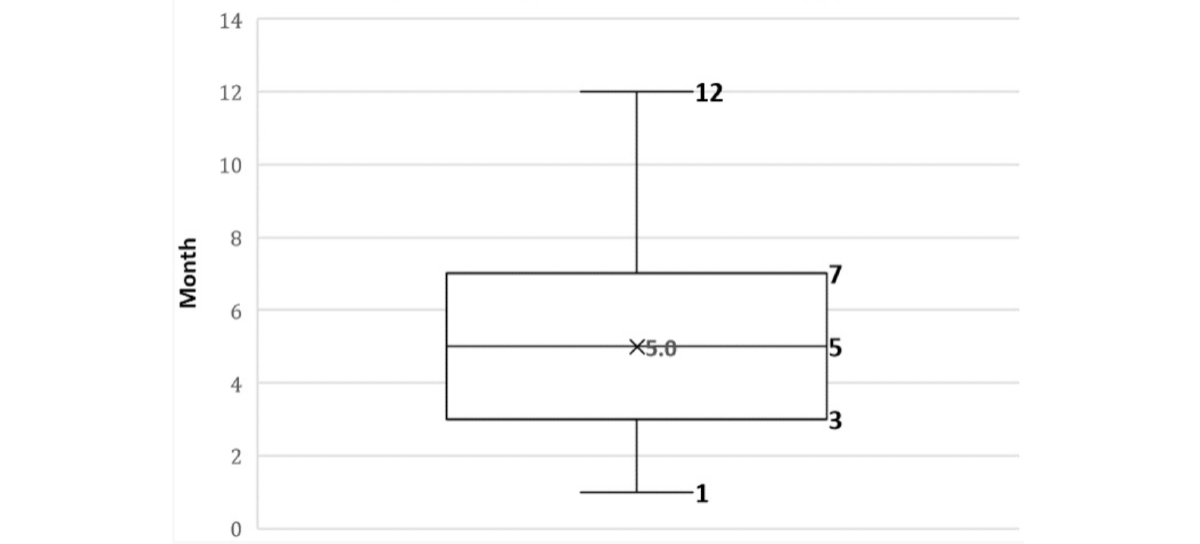
Box and whisker plot of the length of engagement with the Low Carb Program app.

Of the recruited participants (n=105), all enrolled onto the Low Carb Program app. Overall, 90 of the 105 participants (86%) completed the Low Carb Program app registration process and engaged with the Low Carb Program app program. A total of 88 participants (84%) actively engaged with the Low Carb Program app within the previous 30 days. Only a minority of participants (19/105, 18%) completed the entire Low Carb Program app program (defined as completing ≥9 of the 12 education modules available). A total of 58 of the 105 recruited participants (55%) self-reported outcomes from the Low Carb Program app. Of the 47 participants who did not self-report outcomes, 29 participants (62%) were actively using the Low Carb Program app at follow-up assessments. The mean duration between baseline (registration) and follow-up check-in to self-report HbA_1c_ and body weight was 5 months. Half of all participants engaged with the Low Carb Program app between 3 and 7 months (range 1-12 months), as shown in Figure 2. Mean duration of clinical follow-up for recruited participants who received the Low Carb Program app was 7.4 months.

### Clinical Effectiveness

Paired data were available from 48 Low Carb Program app users for body weight and 41 Low Carb Program app users for HbA_1c_. Paired sample *t* test analysis revealed a statistically significant mean loss of body weight of 2.7 kg (*P*=.001) and improvement in HbA_1c_ of 3.3 mmol/mol (*P*=.01), summarized in Table 3. The mean percentage weight loss of the whole cohort was 2.5%, with 10 of 48 patients (20.8% of our sample) achieving weight loss of more than 5% and 18 of 48 patients (23% of our sample) achieving weight loss of at least 3%.

**Table 3:** Summary of data for paired Student *t* test comparisons between baseline and follow-up.

Baseline phenotype	Baseline Low Carb Program app users, mean (SD)	Follow-up Low Carb Program app users, mean (SD)	Number of patients, n	Mean difference (95% CI)	*P* value
Body weight (kg)	132.7 (30.7)	130.0 (31.9)	48	–2.7 (–4.3 to –1.1)	.001
HbA_1c_ (mmol/mol)	53.3 (15.8)	50.0 (13.4)	41	–3.3 (–5.7 to –0.8)	.01

### Adverse Events

No adverse events were reported throughout the duration of this study.

### Comparison With Prepandemic Control Group

Data comparisons between the Low Carb Program app user group and the pre–COVID-19 retrospective control group (usual clinical care) revealed similar loss of body weight and change in HbA_1c_ between the two groups (Table 4). The mean percentage weight loss in the control group was 0.88%, with 15 of 92 (16.3%) patients achieving weight loss of more than 5%. This is in keeping with the statistical test that showed that there was no statistically significant difference in the weight loss between the app group and retrospective group. Table 4 shows a summary of data for independent Student *t* test comparisons between the Low Carb Program app user group and the retrospective control group.

**Table 4:** Summary of data for independent Student *t* test comparisons between the Low Carb Program app user group and retrospective control group.

Baseline phenotype	Mean change in control group (SD), n^a^	Mean change in Low Carb Program app group (SD), n^a^	Mean difference in 6-month outcome between Low Carb Program app user group and control group (95% CI)	*P* value (equal variances not assumed)
Body weight (kg)	–1.1 (6.5), n=92	–2.7 (5.5), n=48	1.7 (–0.4 to 3.7)	.12
HbA_1c_ (mmol/mol)	–0.5 (11.9), n=87	–3.3 (7.7), n=41	2.7 (–0.7 to 6.2)	.12

^a^Indicates the number of patients for whom the outcome of interest was available.

## Discussion

### Principal Results

This study demonstrated the feasibility of using the Low Carb Program app as part of a remotely delivered obesity multidisciplinary team during the COVID-19 pandemic era, as well as efficacy regarding both loss of body weight and improvement in glycemic control over a 7-month period.

Our data confirm equivalence of body weight reduction resulting from remote obesity management complemented by digitally enabled support through the Low Carb Program app versus traditional face-to-face hospital-based obesity management implemented during the pre–COVID-19 era. This is a novel insight that has important implications for the future delivery and hybridized digitalization of hospital-based obesity services. Our data corroborate a recent meta-analysis on mobile app interventions, showing similar reductions in body weight and improvements in glycemic control [16]. The clinical relevance of this study is the impact of digital tools on weight loss and the need to incorporate them into the NHS obesity pathways. Meaningful improvements in blood glucose levels and dyslipidemia are seen with a weight loss of 3% or more [21,22]. In our cohort, 23% (18/48) had weight loss of over 3%, and this will translate into meaningful clinical improvements in blood glucose, as well as improvements in dyslipidemia. Moreover, we should not underestimate the utility of any weight loss on the emotional and psychological status of people who struggle with their weight. This can have great motivational effects, and the fact that this was “self-induced,” without any active input from a dietitian, means it is likely to also improve self-esteem and self-confidence, which in turn should help to encourage further weight loss over time.

Most oral therapies for glycemic control in patients with T2D only lower HbA_1c_ by between 5 and 10 mmol/mol. In our study, we observed an average reduction of 3 mmol/mol, which is almost on par with some oral therapies for T2D management. The importance of good glycemia control was highlighted in the UK Prospective Diabetes Study, as it reduces the risk of developing diabetes complications [23]. The improvement achieved by using the Low Carb Program complements the effects of other traditional therapies for glycemia, and therefore enables more patients with diabetes to get within a target HbA_1c_ level.

It is important to highlight the serendipitous nature of our study, with its conception and initiation prior to the emergence of COVID-19 onto the world stage. Although originally designed as a means to complement the traditional standard of face-to-face, multidisciplinary management of obesity delivered within a hospital-based setting, use of the Low Carb Program app instead complemented the remote delivery of obesity management delivered over the telephone by a diminished team (without focused dietetic support), due to the obstructive effects of the COVID-19 pandemic. Thus, the execution of our study morphed out of a necessity to adapt and align our clinical practice in response to a global pandemic, in combination with a study design in which COVID-19 did not feature. In retrospect, the Low Carb Program app seems more apt to complement a remote care model than one of a traditional (pre–COVID-19) standard of care. Furthermore, during the COVID-19 pandemic, none of our patients received any dietetic support due to staff redeployment at UHCW. Therefore, we cannot attribute the changes in body weight and HbA_1c_ observed with the Low Carb Program app and remote management to any dietetic input. Conversely, these clinical outcomes stemmed from remote medical and psychological input combined with patient engagement with the Low Carb Program app (including learned education, knowledge, and lifestyle behavioral and dietary changes).

The Low Carb Program app had a high sign-up rate, with all participants signing up (105/105, 100%), and a high engagement rate at follow-up (88/105, 83.8%). Overall, 15 of the 105 participants (14.3%) did not complete the registration process and 47 of the 105 participants (44.8%) did not report outcomes once they had activated their registration. However, much of this group (29/47, 61.7%) were actively using the app at follow-up. These results suggest that the intervention requires adaptations to fully engage patients diagnosed with obesity and that other features of the app may be more engaging than health tracking.

The emergence of digital health care interventions that complement conventional health care delivery (traditional face-to-face or remote) provides an opportunity to personalize the treatment and care provided to patients [24,25]. The implementation of our Low Carb Program app enabled a novel, multidimensional, holistic approach to weight management [26], with the convenience and accessibility of social, dietary, and psychological support. Remote self-monitoring and self-reporting of body weight provides the potential for useful feedback to relevant health care professionals to enable prioritization of health care resources and thereby facilitate efficient health care delivery. The implementation of digital interventions in the context of weight management services also has the potential to improve the overall personal experience of patients to facilitate successful weight loss and bridge communications with relevant health care professionals.

Furthermore, the integrated use of digital health care technology within established clinical pathways aligns with NHS priorities, such as supporting the prevention of obesity-related disease and the transition toward digitally enabled health care [10]. The current literature provides supportive evidence for the popularity of self-monitoring tools [27], interactivity with other users [28] and messages enabling frequent check-ins [29] among patients attending weight management services generally. A recent narrative review on the use of digital technology in the context of weight management concluded that while evidence exists to support the usefulness of digital interventions in obesity management, this field remains largely unexplored [30], with a lack of acknowledgment of the role of digital tools within national policies [31]. Further research should explore the usability of the Low Carb Program app, the reasons for nonengagement among some participants, and the features that participants find useful. It is also important to highlight a potential for social inequity when using digital tools for those who cannot afford or access digital technologies, and the importance of trying to minimize barriers to access of digital tools among potential users. That said, the app provides support for “hard-to-reach” communities in the United Kingdom including South Asian populations that speak Punjabi, Hindi, Gujarati, Urdu, and Bengali. Although a physical “starter pack” was available for digitally excluded patients containing printed copies of education, recipe cards, and coaching by phone, nearly all patients in the clinic had a smartphone.

### Limitations

Our study had several limitations. Due to the observational nature of our study, and its design as a clinical innovation, there was no randomization and no inclusion of a control (placebo) group for direct comparison with those participants who engaged with the Low Carb Program app in combination with remotely delivered obesity management. Although it is possible that the clinical benefits of this combination stemmed solely from the remote interactions with members of the obesity team, this seems unlikely given the notable absence of any focused support from specialist dieticians. A much more likely scenario is that engagement with the Low Carb Program app helped to complement remote management in patients’ achievements of clinical outcomes. Furthermore, although we did not include a control group, we did make comparisons retrospectively with a group of patients who had received a traditional standard of care pre–COVID-19.

As this study assessed the feasibility of the app, we did not collect information on medication changes that future studies should capture. Change in glycemic therapy could be a confounder, given the effects of SGLT2 inhibitors and GLP1 analogues on body weight. Additionally, data on BMI was not available for all participants and therefore we did not include it as part of this pilot study.

A further limitation included a lack of data collection on all the patients originally invited to use the Low Carb Program app. These data would have been useful to understand why some patients declined to register with the Low Carb Program app. Due to the impact of the COVID-19 pandemic and the requisite remote management paradigm, we were not able to measure participants’ body weight using the weighing scales in our clinical setting. Rather, all participants self-measured and self-reported their body weight measurements throughout. This may have introduced some inaccuracy, and we were not able to verify self-reported body weights.

Another potential confounder includes the unusual scenario of the national “lockdown” within the United Kingdom due to the COVID-19 pandemic that coincided with much of the study period. However, given the documented propensity for weight gain during the lockdown and self-quarantine period [32-34], this insight further promotes our own data of body weight loss as even more remarkable. Finally, there was anonymization of participant self-reporting of data through the Low Carb Program app. Therefore, it was not possible to match these self-reported data with clinically derived data for each participant due to the design of the Low Carb Program app. Future studies should explore the requisite adaptations for the Low Carb Program app to facilitate its broader application within the clinical arena.

Patient engagement with the app at follow-up was high. This may be because patients received the app at no cost to them. Although this may impact engagement with the app, in the United Kingdom, costs of therapy are borne by the state health care provider and given to patients for free, which reflects the real-world nature of this study. Future studies should evaluate the impact of the app based on the referral mechanism to compare whether paying for access to the app or receiving it for free affects app usage and outcomes.

### Conclusion

We provide the first evidence to support the feasibility of implementing the Low Carb Program app combined with remote obesity management, providing the first proof of concept for digitalized management within a hospital-based (tier 3) obesity service. We demonstrate the clinical efficacy of such an approach, both regarding loss of body weight and improvement in glycemic control. Future studies should explore how to adapt the Low Carb Program app to populations seen within the clinical obesity setting to improve user engagement and long-term outcomes. Ultimately, the Low Carb Program app represents a management option that is potentially both accessible and scalable at the population level. Furthermore, from a preventive perspective, the Low Carb Program app has relevance for the general population, regardless of obesity status. A healthy lifestyle is important for all of us. After all, an ounce of prevention is worth a pound of cure.

## Abbreviations

T2D: type 2 diabetes
NHS: National Health Service
UHCW: University Hospitals Coventry & Warwickshire
